# Computing inelastic neutron scattering spectra from molecular dynamics trajectories

**DOI:** 10.1038/s41598-021-86771-5

**Published:** 2021-04-12

**Authors:** Thomas F. Harrelson, Makena Dettmann, Christoph Scherer, Denis Andrienko, Adam J. Moulé, Roland Faller

**Affiliations:** 1 Department of Chemical Engineering, University of California-Davis, 1 Shields Ave, Davis, CA 95616 USA; 2 Molecular Foundry, Lawrence Berkeley National Laboratory, 1 Cyclotron Road, Berkeley, CA 94720 USA; 3 Department of Materials Science and Engineering, University of California-Davis, 1 Shields Ave, Davis, CA 95616 USA; 4 Max Planck Institute for Polymer Research, Ackermannweg 10, 55128 Mainz, Germany

**Keywords:** Materials for energy and catalysis, Theory and computation, Materials science, Organic molecules in materials science

## Abstract

Inelastic neutron scattering (INS) provides a weighted density of phonon modes. Currently, INS spectra can only be interpreted for perfectly crystalline materials because of high computational cost for electronic simulations. INS has the potential to provide detailed morphological information if sufficiently large volumes and appropriate structural variety are simulated. Here, we propose a method that allows direct comparison between INS data with molecular dynamics simulations, a simulation method that is frequently used to simulate semicrystalline/amorphous materials. We illustrate the technique by analyzing spectra of a well-studied conjugated polymer, poly(3-hexylthiophene-2,5-diyl) (P3HT) and conclude that our technique provides improved volume and structural variety, but that the classical force field requires improvement before the morphology can be accurately interpreted.

## Introduction

Accurate models of local dynamics in amorphous materials remains a challenge in several fields of scientific research. An improvement in the assignment of localized dynamics for different molecular environments would have major impacts across many disciplines^1^. Specific examples of material classes for which accurate calculations yielded key insights into the underlying physical properties include polymers^2–4^, metal-organic frameworks^5–7^, lipid membranes^8^, and proteins^9,10^. Currently, a robust method, based on density functional theory (DFT), exists for modeling local dynamics in crystalline materials. However, because most real materials are semicrystalline or amorphous, the DFT method is too computationally costly to simulate complex morphologies (scales as $\sim N_e^3$; where $N_e$ is the number of electrons). Alternatively, there is a molecular dynamics (MD) based method which has been used to assign local dynamics, which scales much better than DFT (${\mathcal {O}}(N_a\log N_a)$ or ${\mathcal {O}}(N_a)$ for Ewald-based or cutoff-based methods, respectively, where $N_a$ is the number of atoms), but relies on some troubling assumptions^11^. In that method, the isotropic approximation is made, and mean-squared displacements of each atom are proportional to temperature. This leads to a violation of the uncertainty principle when the frequency of the motion is greater than the equivalent temperature of the system ($\hbar \omega > k_BT$). Therefore, we propose a new method to interpret local dynamical data that has reduced computational cost relative to the DFT method but improves on the assumptions of the standard MD method.

When designing a model for dynamics, it is crucial that the predictions of the model be validated experimentally. While there are several experimental techniques to study local dynamics, inelastic neutron scattering (INS) stands apart. INS experiments provide valuable information on the structure and dynamics of molecular systems such as polymers^2,4^, metal-organic frameworks^7^, molecular crystals^12,13^, and crystalline oxide materials^14^. Unlike optical methods such as FTIR and Raman, INS has no selection rules based on molecular symmetry, meaning all overtones are observable^11^. This increases the density of information in the spectra. In addition, INS experiments detect atomistic motions in the 1–1000 cm$^{-1}$ energy range, which is higher in energy than the slow diffusive motions probed in quasi-elastic neutron scattering and neutron spin echo experiments. All these properties make INS the most accurate and comprehensive technique for measuring local dynamics in the 1-1000 cm$^{-1}$ energy range.

INS spectrometers can be generally divided into those with direct or indirect geometries (with some exceptions such as LAGRANGE at ILL^15^ which utilizes both analyzer crystals and a monochromator). Direct geometry experiments provide the relationship between the frequency of the dynamics and the wavevector of the dynamics (e.g. phonon dispersion relationships), while most indirect spectrometers capable of performing vibrational spectroscopy trade dispersion relationships for increased count rates, which boosts the signal-to-noise ratio. There are examples of indirect spectrometers that can determine the $q{-}\omega$ relationships of the dynamic structure factor, such as BASIS at ORNL^16^, but these instruments typically cannot reach the required energy transfers for vibrational spectroscopy. For materials containing significant amounts of $^1$H, the information about the relationship between the wavevector and frequency is destroyed due to incoherent scattering processes, which makes indirect spectrometers the preferred measurement for protonated organic materials. The VISION spectrometer at Oak Ridge National Laboratory is an inverted geometry, time-of-flight spectrometer that has higher count rates, higher energy resolution, and higher dynamic range than other similar instruments^17^. Resolution of the high energy dynamics is improved by cooling of the sample to cryogenic temperatures to reduce the Debye–Waller factor. Thus, our new methodology is aimed at simulating the output of a low-temperature experiment performed on the VISION spectrometer or similar spectrometers.

In this paper, we develop a technique for simulating INS spectra for glassy organic materials. We demonstrate a novel method for transforming MD trajectories into theoretical INS spectra which, unlike the established technique, can predict overtones and does not rely on the isotropic approximation.

## Theory

**Figure 1:**
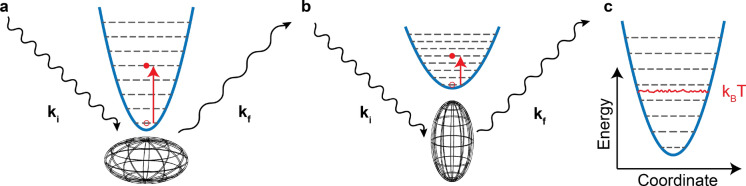
(**a**,**b**) Schematic depicting neutron scattering off a prolate anisotropic oscillator (oval wireframe object) in two different orientations demonstrating how molecular orientation changes the available scattering processes. In (**a**) the momentum transfer is directed solely along the short axis of the oscillator, creating vibrationally excitations along that axis. In (**b**) the oscillator is rotated such that the momentum transfer is directed along the long axis of the oscillator, which has smaller spacing between energy levels. (**c**) Potential energy surface for a harmonic oscillator. The quantum mechanical levels are represented by grey dashed lines, and the rough red line represents a trajectory of a classical simulation.

Developing a method for computing the inelastic neutron scattering function from time-ordered trajectories is challenging because the underlying scattering physics depends on quantum mechanical information about the dynamics of the system. This is seen in the low-temperature form of the scattering function, which is,

1 $$\begin{aligned} S\left( \vec {q},\omega \right) = \sum _{n,i,j} \sigma _i\frac{\left( \vec {q}\cdot \vec {u}_{ij}\right) ^{2n}}{n!} \exp \left[ -\sum _j \left( \vec {q}\cdot \vec {u}_{ij}\right) ^2\right] \delta \left( \omega -n\omega _j\right) \end{aligned}$$

where $\vec {q}$ is the momentum transfer vector, $\omega$ is the frequency of oscillation, $\sigma _i$ is the neutron cross section for atom *i*, $\vec {u}_{ij}$ is the quantum-mechanical ground state displacement of mode *j* projected onto atom *i*, and *n* is the quantum number of the *n*-th excitation. The exponential term in the scattering function is called the Debye–Waller factor. The probability that the neutron interacts with atom *i* is represented by Eq. (1), in which $\hbar \vec {q}$ momentum ($\vec {q}$ is the difference between $\vec {k}_i$ and $\vec {k}_f$ in Fig. 1) is transferred to the atom causing an excitation of the vibrational state of the *j*-th oscillator to the *n*-th overtone. Figure 1a demonstrates the physics of an inelastic neutron scattering event represented by the scattering function in Eq. (1): a neutron scatters off of an anharmonic oscillator (which models the atomic dynamics), transferring energy to the atom by exciting one (or several) of its vibrational modes, and exiting the sample toward the detector with a smaller wavevector. The orientation of the molecule relative to the direction of the incoming neutron is important as most atoms in a molecular solid do not vibrate isotropically. Rotation of an anisotropic oscillator changes the probabilities of excitation for all of the modes. In specific orientations, some modes are completely invisible to neutron scattering because the direction of oscillation is perpendicular to the momentum transfer of the scattering event (an example is shown in Fig. 1b). This phenomenon is mathematically represented by the scalar products between $\vec {q}$ and $\vec {u}_{ij}$ in Eq. (1). Therefore, we see that computing a scattering function requires computation of the ground state displacement of mode *j* projected onto atom *i* ($\vec {u}_{ij}$) and its corresponding frequency ($\omega _j$).

The most common method for computing these parameters uses DFT simulations combined with normal mode analysis^18^. The $\vec {q}$ dependence of the scattering function is assumed to follow that of inverted geometry neutron spectrometers^11^, such as VISION^17^, for which $q^2\propto \omega$. The exact equations for *q* as a function of $\omega$ are determined by the instrument setup (e.g. position and type of analyzer crystals). It is also possible to use a classical forcefield in place of a density functional to calculate the dynamical matrix required to obtain the phonon eigenvectors and frequencies, which allows the simulation of phonons for systems larger than DFT, which can typically handle ($\ge 1000$ atoms).

While normal mode analysis is useful for comparing DFT simulations to INS spectra, it becomes unfeasible for large systems encountered in MD simulations because of the computational requirements for diagonalizing matrices of dimension three times the number of atoms in the simulation. To decrease the computational cost, previous studies comparing MD simulations to INS spectra have computed the Fourier transform of the velocity autocorrelation function (i.e. the power spectrum)^2,19^. Recently, there has been some work using autocorrelation functions of MD simulations to obtain phonon dispersion curves^20,21^. These methods share a lot theoretical similarities to our approach, but are limited to calculating dispersion curves for crystalline systems, which are observed from angle-resolved coherent neutron spectra (e.g. from the SEQUOIA spectrometer^22^). In addition to being more computationally expensive to simulate, most organic materials contain hydrogen, which means INS experiments yield an incoherent signal without a well-defined dispersion curve. To simulate the incoherent INS spectra for heterogeneous materials involves calculating the phonon density of states (pDOS) from the velocity autocorrelation function. The pDOS is then projected onto specific atom types that strongly scatter neutrons (typically $^1$H) and compared directly to INS spectra^2^. This method leads to inaccuracies, as the pDOS is only part of the input into the calculation of an INS spectrum from the scattering function (Eq. 1). The pDOS does not contain the Debye–Waller factor, the $\vec {q}$ dependence, or overtones, which means that the projected pDOS can only be qualitatively compared to INS data. The inaccuracy of the pDOS method is demonstrated in Fig. 1c, which shows the energy profile of a classical trajectory of a harmonic oscillator thermostatted to temperature *T*. Each classical mode (represented by an oscillator) would contribute to a peak at the correct frequency, but the height of the peak is proportional to *T*, which is not consistent with Eq. (1). The formulation for computing INS spectra from MD trajectories with the least number of approximations has been discussed previously^11^ (e.g. overtones, Debye–Waller factor, and $\vec {q}$ dependence), but this method still leads to inaccuracies due to the lack of frequency dependence of the anisotropic vibrational disorder, and quantum ground state atomic displacements. In this manuscript, we present a method for computing INS spectra from all-atom MD trajectories that is as correct as the normal mode-based methods used in conjunction with DFT simulations.

Energy resolved dynamics from MD simulations are characterized by the power spectrum, which is the Fourier transform of the velocity autocorrelation function,

2 $$\begin{aligned} v_i^2(\omega ) = F\left\{ \langle \vec {v}_i(t)\cdot \vec {v}_i(t+\tau )\rangle \right\} \end{aligned}$$

in which $v_i$ is the velocity of atom *i*, $F\{...\}$ represents a Fourier transform, and $\langle ...\rangle$ represents the time average. The power spectrum can be related to the eigenvectors (normal modes) and eigenvalues. To show this, we first expand the atomic velocities into contributions from the normal modes,

3 $$\begin{aligned} \vec {v}_{i}(t) = \sum _j c_{ij}\vec {\nu }_j(t) \end{aligned}$$

where $\nu _j$ is the velocity of mode *j* along the vibrational mode coordinate, and $c_{ij}$ is the unitary transformation matrix between the atomic basis and the vibrational basis. Even though it is possible to construct the power spectrum from the phonon eigenvectors, it is not generally possible to reconstruct the eigenvectors from the power spectrum because of potential degeneracies in the eigenspectrum.

We are assuming a harmonic approximation, which means that the velocity of the classical oscillators, $\vec {\nu }_j$, takes the form,

4 $$\begin{aligned} \vec {\nu }_j(t)=\vec {V}_j \cos \left( \omega _j t + \delta _j\right) \end{aligned}$$

where $\vec {V}_j$ is the maximum velocity of mode *j*, $\omega _j$ is the frequency, and $\delta _j$ is the phase. This equation implies that the power spectrum of $\vec {v}_i$ using the expansion in Eq. (3) reveals independent oscillators at different frequencies,

5 $$\begin{aligned} v_i^2(\omega ) = \sum _j \frac{\left. \pi |c_{ij}\right| ^2V_j^2}{2} \delta \left( \omega -\omega _j\right) \end{aligned}$$

in which $|c_{ij}|^2$ is the probability that atom *i* participates in mode *j*. The power spectrum cannot differentiate between different modes at the same frequency. This represents a potential problem in the evaluation of strength of the vibrational eigenvectors from the power spectrum, as the change in phase between any pair of oscillators ($\delta _j$ in Eq. 4) impacts the observed peak height in the power spectrum. However, in Eq. (5) we are asserting that the oscillators are uncorrelated, which is true if the simulation has at least a weak thermostat (or weak anharmonicities) that decorrelates the oscillators. Then the relative phase differences between the oscillators average to zero, and the observed peak heights of the power spectrum are equal to the sum of each of the contributing vibrational eigenvectors. Further details, including the relationship between the above equations and the computed objects in the code, are available in SI Section S3.

The accuracy of simulated phonons is assessed through comparison to experimental INS spectra. Low temperature INS spectra are well described by the scattering function in Eq. (1)^11^. The quantum mechanical displacements ($\vec {u}_{ij}$) are not directly accessible from the atomic power spectra in MD trajectories, so we seek a connection. The magnitude of the ground state displacement of a quantum harmonic oscillator is given by,

6 $$\begin{aligned} u_{ij}^2\left( \omega _j\right) = \frac{|c_{ij}|^2\hbar }{2\mu _j \omega _j} \end{aligned}$$

where $\mu _j$ is the reduced mass of mode *j*^23^. Note that every term in Eq. (6) is accessible from the atomic power spectra, except $\mu _j$. To find $\mu _j$ from information in the power spectra, we use the equipartition theorem:

7 $$\begin{aligned} k_BT = \mu _j \langle \nu _j^2\rangle = \mu _j \frac{V_j^2}{2} \end{aligned}$$

Simple rearrangement of (5) to (7) provides a relationship for $u^2_{ij}$ as a function of the power spectrum:

8 $$\begin{aligned} u_{i}^2(\omega ) = \frac{\hbar v_i^2(f)}{\omega k_BT} \end{aligned}$$

where *f* is the same as $\omega /(2\pi )$, and we have implicitly summed over all degenerate modes at a given frequency, $\omega$, to remove the dependence on the *j* index. Complete details of this derivation are available in SI Section S1. Essentially, the knowledge of the effective maximum velocity of modes at frequency $\omega$ provides the curvature of the potential energy surface shown in Fig. 1c, which is directly related to the ground state displacement of a quantum mechanical object acting on the same potential energy surface (assuming the harmonic approximation holds).

The units of Eq. (8) are not the same as Eq. (6) as $v_i^2(\omega )$ has units of $m^2/s$ instead of $m^2/s^2$ because of the action of the Fourier transform. The correct units are recovered with an integral over $\omega$. Thus, Eq. (8) contains both $u_{i,j}$ and the delta function from the scattering law (Eq. 1) because the delta function, $\delta (\omega -\omega _j)$ has units of $\omega ^{-1}$. This formulation of $u_i^2(\omega )$ can be used in the scattering law under the assumption that all the oscillators have an isotropic displacement from their equilibrium positions, which is generally untrue. Thus, the scattering rate into a particular solid angle depends on the momentum scattering vector, $\vec {q}$, in Eq. (1).

For inverted geometry spectrometers, such as VISION at ORNL or TOSCA at ISIS, the magnitude of the momentum scattering vector changes with energy transfer, meaning the instruments are not well-equipped for independently probing both the $\vec {q}$ and $\omega$ dependences of the scattering law. In addition, it is experimentally difficult to obtain single crystals that are large enough to obtain good neutron scattering data ($\sim 1$ g is needed^11^). Thus, crystalline powder samples are commonly used for neutron measurements, which implies an orientational average over the scattering function. The orientational dependence of the scattering function is graphically depicted in Fig. 1a,b. The equation for the orientational average has no analytical solution, and must be numerically approximated. The most common approach to simplify the powder average is the “almost isotropic” approximation^11,18^. This approach is used to approximate the powder average of the first overtone of the scattering equation ($n=1$ in Eq. 1) which we write as,

9 $$\begin{aligned} S_{0\rightarrow 1}\left( q,\omega _j\right)&= \frac{1}{4\pi }\int S_{0\rightarrow 1}\left( \vec {q},\omega _j\right) d\Omega \end{aligned}$$

10 $$\begin{aligned} S_{0\rightarrow 1}\left( \vec {q},\omega _j\right)&= \sum _{i} \sigma _i\left( \vec {q}\cdot \vec {u}_{ij}\right) ^{2}\exp \left[ -\sum _j \left( \vec {q}\cdot \vec {u}_{ij}\right) ^2\right] \end{aligned}$$

where $S_{0\rightarrow 1}(q,\omega _j)$ is the powder averaged scattering function for the first overtone, and $d\Omega$ is the differential angle. The equation is restricted to a single mode *j* for simplicity; the full scattering function is the sum over all mode contributions. The almost isotropic approximation^11^ changes this equation to:

11 $$\begin{aligned} S_{0\rightarrow 1}\left( q,\omega _j\right)\approx & {} \sum _i \frac{q^2}{3}{{\,\mathrm{Tr}\,}}\left( \overline{\overline{B}}_{ij}\right) \exp \left( -q^2\alpha _{ij}\right) \end{aligned}$$

12 $$\begin{aligned} \alpha _{ij}= & {} \frac{1}{5}\left[ {{\,\mathrm{Tr}\,}}\left( \overline{\overline{A}}_i\right) + 2\left( \frac{\overline{\overline{B}}_{ij} : \overline{\overline{A}}}{{{\,\mathrm{Tr}\,}}\left( \overline{\overline{B}}_j\right) }\right) \right] \end{aligned}$$

13 $$\begin{aligned} \overline{\overline{B}}_{ij}= & {} \vec {u}_{ij} \vec {u}_{ij}^T \end{aligned}$$

14 $$\begin{aligned} \overline{\overline{A}}_i= & {} \sum _j \overline{\overline{B}}_{ij} \end{aligned}$$

where $\overline{\overline{B}}_{ij}$ is defined as the displacement tensor of atom *i* for mode *j*, and $\overline{\overline{A}}_i$ is the total displacement tensor for atom *i*. In prior studies^11,24^, calculation of the first overtone uses Eq. (11,12,13,14), while higher overtones are usually computed within the isotropic approximation as the almost isotropic approximation only marginally improves accuracy while severely complicating the computation as discussed previously^11^. The isotropic approximation of the Debye–Waller factor is

15 $$\begin{aligned} {\text {DWF}} = \exp \left[ -q^2A_i/3\right] . \end{aligned}$$

To make our formulation consistent with the almost isotropic approximation, we must create analogous versions of $\overline{\overline{A}}_i$ and $\overline{\overline{B}}_{ij}$ from MD trajectories. The power spectrum in Eq. (8) is analogous to the trace of $\overline{\overline{B}}_{ij}$, so we expand the power spectrum to include all possible cross correlations between the different Cartesian components of the velocity. Thus, we define an outer-product velocity correlation function, $\langle \vec {v}^{\,*}_{i}(t) \vec {v}^{\,T}_{i}(t+\tau ) \rangle$, which is a Hermitian tensor, and its corresponding Fourier transform is shown in matrix form in Eq. (16).

16 $$\begin{aligned} \vec {v} \vec {v}^T(\omega ) = \begin{pmatrix} \langle \left| v_x(\omega )\right| ^2 \rangle &{} \langle v_x^*(\omega )v_y(\omega ) \rangle &{} \langle v_x^*(\omega )v_z(\omega ) \rangle \\ \langle v_y^*(\omega )v_x(\omega ) \rangle &{}\quad \langle \left| v_y(\omega )\right| ^2 \rangle &{}\quad \langle v_y^*(\omega )v_z(\omega ) \rangle \\ \langle v_z^*(\omega )v_x(\omega ) \rangle &{}\quad \langle v_z^*(\omega )v_y(\omega ) \rangle &{}\quad \langle \left| v_z(\omega )\right| ^2 \rangle \end{pmatrix} \end{aligned}$$

We can replace $v_i^2(\omega )$ with $\vec {v} \vec {v}^T(\omega )$ in Eq. (8) to construct an analogous version of $\overline{\overline{B}}_{ij}$, which extends our formulation beyond the isotropic approximation. In this formulation, $\overline{\overline{A}}_i$ is the integral of $\overline{\overline{B}}_{ij}$ with respect to $\omega$. From here, our analogous versions of $\overline{\overline{A}}_i$ and $\overline{\overline{B}}_{ij}$ can be inserted into the almost isotropic approximation equation providing the scattering function of the first overtone completely in terms of the information present in a classical MD simulation.

To summarize, we have computed ground state displacements of a set of quantum harmonic oscillators from the information in the classical power spectrum, and we have approximated the powder average while accounting for anisotropies of vibrational motion. The only remaining problem is the inclusion of overtones in the simulated spectrum because the power spectrum does not contain any overtones in the harmonic approximation. It is possible that overtones appear in highly anharmonic modes, which is why we run the molecular dynamics simulation at low temperature (which also simulates the INS experiment at low temperature). Obtaining the spectral contribution of overtones is not trivial because combination excitations need to be calculated (simultaneous excitation of two different $0\rightarrow 1$ transitions) in addition to the principal overtones ($0\rightarrow 2$ transitions). To obtain the full second overtone spectrum, including combination excitations, the fundamental spectrum (obtained from $\langle \vec {v} \vec {v}^T(\omega )\rangle$) is convolved with itself:

17 $$\begin{aligned} \overline{\overline{B}}_{0\rightarrow 2}(\omega ) = \overline{\overline{B}} *\overline{\overline{B}} = \int \overline{\overline{B}}(\omega ')\overline{\overline{B}}(\omega -\omega ')d\omega ' \end{aligned}$$

In principle, since $\overline{\overline{B}}$ is a matrix, the convolution produces a fourth order tensor that must be contracted into a second order tensor (to be used in the almost isotropic approximation), which we approximate as a simple matrix multiplication between $\overline{\overline{B}}$ with itself at a different frequency. To get the third overtone spectrum, we convolute $\overline{\overline{B}}_{0\rightarrow 2}(\omega )$ with $\overline{\overline{B}}(\omega )$. Therefore, the spectrum of the *n*-th overtone in INS can be computed as the *n*-th convolution of $\overline{\overline{B}}$ with itself. If we define the convolution as an operator, we can show that the series of convolution operators reproduces the intermediate scattering function (see Supplemental Information section S2), which has been used for analyzing all other forms of inelastic neutron scattering experiments (e.g. QENS, neutron spin echo, etc.). The intermediate scattering function is a useful starting point to analyze other dynamical measurements such as X-ray techniques and dynamic light scattering, meaning that our approach can be used for something other than neutron scattering measurements.

The computational workflow is as follows:

1. The atomic displacement tensor, $\overline{\overline{B}}_{0\rightarrow 1}$, for atom *i* was computed from the molecular dynamics trajectory for that atom using Eqs. (8) and (16).
2. The vector containing the $q^2$ dependence as a function of $\omega$ was computed (specific to the back scattering analyzers of the VISION spectrometer^17^),
3. The Debye–Waller factor was computed for both the isotropic and “almost isotropic” approximations.
4. The $q^2$ vector, the trace of the current $\overline{\overline{B}}_{n}(\omega )$ object (trace of this object is a vector), and the Debye–Waller factor were element-wise multiplied, and the resulting vector was multiplied by the prefactor scalar that depends on the overtone number (same as the loop counter).
5. The resulting vector was added to a total scattering function vector (initialized with zeroes).
6. The current $\overline{\overline{B}}_{n}$ object was convolved with the original atomic displacement tensor, $\overline{\overline{B}}_1$, which generates a new object, $\overline{\overline{B}}_{n+1}$, that corresponds to the next overtone spectrum to be computed in the next iteration of the loop.
7. The above algorithm was repeated for each overtone (total number of overtones is set at the beginning of the workflow).

We assume that the convolution operation between two matrices of functions can be approximated as another matrix that follows from standard matrix multiplication rules (e.g. $\overline{\overline{B}} *\overline{\overline{B}}=\sum _j B_{ij}*B_{jk}$), ensuring that the resulting matrix is Hermitian. Thus, each convolution operation contracts the inner indices of the resulting rank-4 tensor. The Debye–Waller factor from the “almost isotropic” approximation is used for the first overtone $n=1$, and the isotropic approximation is used for all others. The loop index specific prefactor is assumed to be $\frac{3^{n-2}}{n!5^{n-1}}$ because it is a general relation that closely resembles the known prefactors for the first four overtones for powder averages using either isotropic or “almost isotropic” approximations^18^.

Extending our approach beyond the 0 K approximation to the scattering formula can be accomplished by a single additional convolution between the Boltzmann weighted phonon density of states and the 0 K dynamic structure factor. This is shown below,

18 $$\begin{aligned} \langle S(\omega )\rangle&= \int \rho (\omega '; T) S(\omega -\omega ')d\omega ' \end{aligned}$$

19 $$\begin{aligned} \rho (\omega ;T)&= \sum _a e^{\hbar \omega _a/k_BT}\delta \left( \omega + \omega _a\right) \end{aligned}$$

where $\rho$ is the Boltzmann weighted phonon density of states, *S* is the dynamic structure factor computed at 0 K using the algorithm above, and *a* is the index describing the initial state of the system, which can also be expressed in terms of *k*, *s*, the wavevector and branch number of the phonon, respectively. Explicitly writing out the 1-phonon contribution to the thermal average above yields,

20 $$\begin{aligned} \langle S_{0\rightarrow 1}(\vec {q}, \omega )\rangle = \sum _{a,i,j} e^{\hbar \omega _a/k_BT} |\vec {q}\cdot \vec {u}_{ij}|^2\delta \left( \omega + \omega _a-\omega _j\right) \end{aligned}$$

where *a* is the index for the initial state of the system, *i* is the atom number, *j* is the index of the state the system is scattered into. This is equivalent to a result from a prior work by Maradudin and Fein^25^, which was shown to obey detailed balance; a crucial requirement for the correct observation of the ratio between anti-Stokes and Stokes modes in neutron scattering. The Debye–Waller factor is no longer exactly projected onto the ground state displacements of the harmonic oscillator, and instead the mode contributions to the Debye–Waller factor are taken to be the maximal value of either the ground state displacement or the expectation value of the thermal displacement at a given frequency,

21 $$\begin{aligned} {\text {DWF}} = \int {\text {max}}\left[ \left( \vec {q}\cdot \vec {u}_i(\omega )\right) ^2, \left( \vec {q}\cdot \omega \vec {v}_i(\omega )\right) ^2\right] d\omega \end{aligned}$$

where the DWF corresponds specifically to atom *i*, $\vec {u}_i(\omega )$ comes from Eq. (8), and $\vec {v}_i(\omega )$ is the Fourier transform of the velocity trajectory of atom *i* obtained from the MD simulation.

If we want to extend our approach beyond the harmonic approximation, we must identify anharmonicities in the classical power spectra. The anharmonicities manifest as either (1) overtones in the harmonic power spectrum, or (2) decay rates of periodic motions, which broaden peaks in the power spectrum (typically with a Lorentzian lineshape) . The former arises from anharmonicities along the vibrational mode coordinate (see SI Figure S1b), which can be mathematically represented by coupling between energy levels on the same oscillator. The decay rates of classical motions come from coupling between energy levels on different harmonic modes (see SI Figure S1c). In principle, it is possible to approximate the different anharmonic contributions by fitting the power spectrum to an appropriate model. However, fitting the anharmonic overtones is challenging because it is impossible to differentiate between an overtone and a different mode at the same frequency as the overtone. To simplify our formulation, we only consider coupling between different modes and ignore contributions from anharmonic overtones. If we model the classical dynamics as a set of decaying harmonic oscillators (Eq. 22), then we can compute a set of decay constants ($k_j$) that correspond to frequencies ($\omega _j$).

22 $$\begin{aligned} \langle v_i^2 \rangle = \sum _j V_{ij}^2 e^{i\omega _j t - k_j t} \end{aligned}$$

In the observed INS spectrum, anharmonicities cause a decrease in the energetic difference between overtones, meaning that overtones can no longer be calculated via simple Fourier convolution. The goal is to relate the relaxation rates ($k_j$) to the second order energy correction ($E_j^{(2)}$) found by perturbation theory to correct the overtones. In SI section S3, we derive a natural relationship between the relaxation rate $k_i$ and the second order correction to the harmonic energy levels, $E_i^{(2)}$:

23 $$\begin{aligned} \langle k_i\rangle = -\sum _f \frac{4\langle i|V|f\rangle \langle f|V|i\rangle }{\pi \hbar \left( E_f-E_i\right) } = \frac{4E_i^{(2)}}{\pi \hbar } \end{aligned}$$

Given this connection, we can develop a scheme to correct the convolutions to give the proper energetic spacing for an anharmonic oscillator truncated to third order. Importantly, the decay rates determined from a classical simulation correspond to the thermalized initial state, not the vibrational ground state as is typically assumed for computing INS spectra. Correcting for this effect would require multiple classical simulations at different temperatures. While such an implementation is possible, we do not find it practical for large systems of atoms with complex INS spectra, as the harmonic approximation appears to be sufficient.

## Results

**Figure 2:**
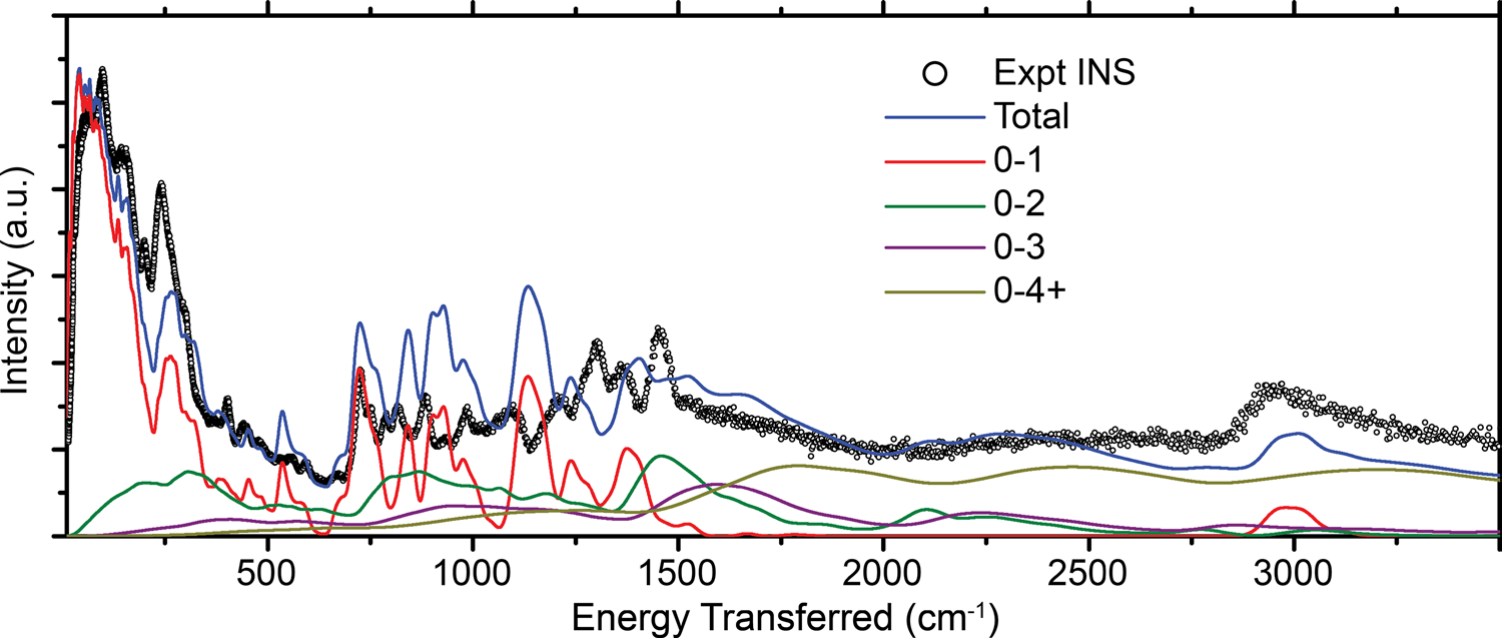
Comparison between experimental INS spectrum for crystalline P3HT (defined as the difference between the semicrystalline spectrum and 55% of the amorphous spectrum) and a simulated spectrum from molecular dynamics. The spectral contributions for individual overtones are also shown. The MD spectrum was convolved by a variable width Gaussian with $\sigma =$ 0.01E, where E is the energy transfer given by the values on the horizontal axis of the plot.

To demonstrate our new approach, we chose to study a semiconducting polymer called P3HT, because it has a complex microstructure owing to its semicrystalline nature and because we previously showed that DFT simulations do not capture low energy INS modes^4^. This makes it challenging to experimentally characterize the microstructure and model the functional properties^3,26^. We used the method presented above to compute an INS spectrum from an MD trajectory and compared to our previously published experimental INS spectrum for semicrystalline P3HT^4^. Since P3HT is known to have amorphous and crystalline regions^26^, we perform independent simulations on both phases, and compare to combinations of regioregular (semicrystalline) and regiorandom (fully amorphous) INS spectra. RRa P3HT is a surrogate for the amorphous regions of regioregular (RR) P3HT because the random head/tail orientation of the monomers increases dihedral distortions through steric interactions between neighboring side-chains. The increased dihedral distortions reduce favorable $\pi$–$\pi$ stacking/crystalline morphologies.

To simulate the crystalline phase of P3HT, we constructed a box of 16 20-mers created from a larger crystalline structure taken from a prior study^27^ that contained 400 chains that was annealed at 300 K and 1 bar for 15 ns. The classical force-field used was taken from Poelking et al.^3^, in which the monomer-monomer torsional parameters and partial charges were parametrized from DFT simulations, and standard OPLS-AA forcefield parameters were used for all other values. The lattice parameters for the room temperature crystalline structure agree with prior experimental work^28^. The structure was quenched down to 10 K, and equilibrated for 10 ns, enough time to allow the kinetic temperature to reach 10 K. We were not interested in finding the thermodynamically equilibrated 10 K structure, as the real sample used in the INS experiment was quickly cooled to cryogenic temperatures with liquid helium, kinetically trapping the configurations during the cooling process. After the short equilibration run at low temperature, a production run was started in which we collected velocity data every 2 fs for 100 picoseconds. Such a high data collection rate is needed to resolve higher energy peaks (C–H stretch period is $\sim 10$ fs). If smaller data collection rates are used, the higher energy peaks will not be resolvable as their frequencies are greater than half of the data collection rate. These high energy peaks are not filtered out of the spectrum, and instead fold back onto the observed spectrum at a different energy, creating unphysical spectral artifacts that contaminate the computed INS spectrum. The choice of 100 ps simulation time leads to a frequency spacing of 0.33 cm$^{-1}$, which is small enough to resolve any salient features in the experimental INS spectrum. One is free to simulate longer, but the high data collection rate means that the file sizes can become extremely large for systems with many atoms. Further details for all MD simulations are in the Methods section.

The resulting simulated spectrum is compared against the experimental INS spectrum in Fig. 2. In addition to the total spectrum, we show the different contributions from each of the overtones. Without any overtones, the simulated spectrum would be equal to the 0-1 spectrum, leading to inaccuracies when comparing to the experimental data. Properly accounting for the overtones allows for quantitative comparisons between theory and experiment, which has applications in direct forcefield parameter fitting.

**Figure 3:**
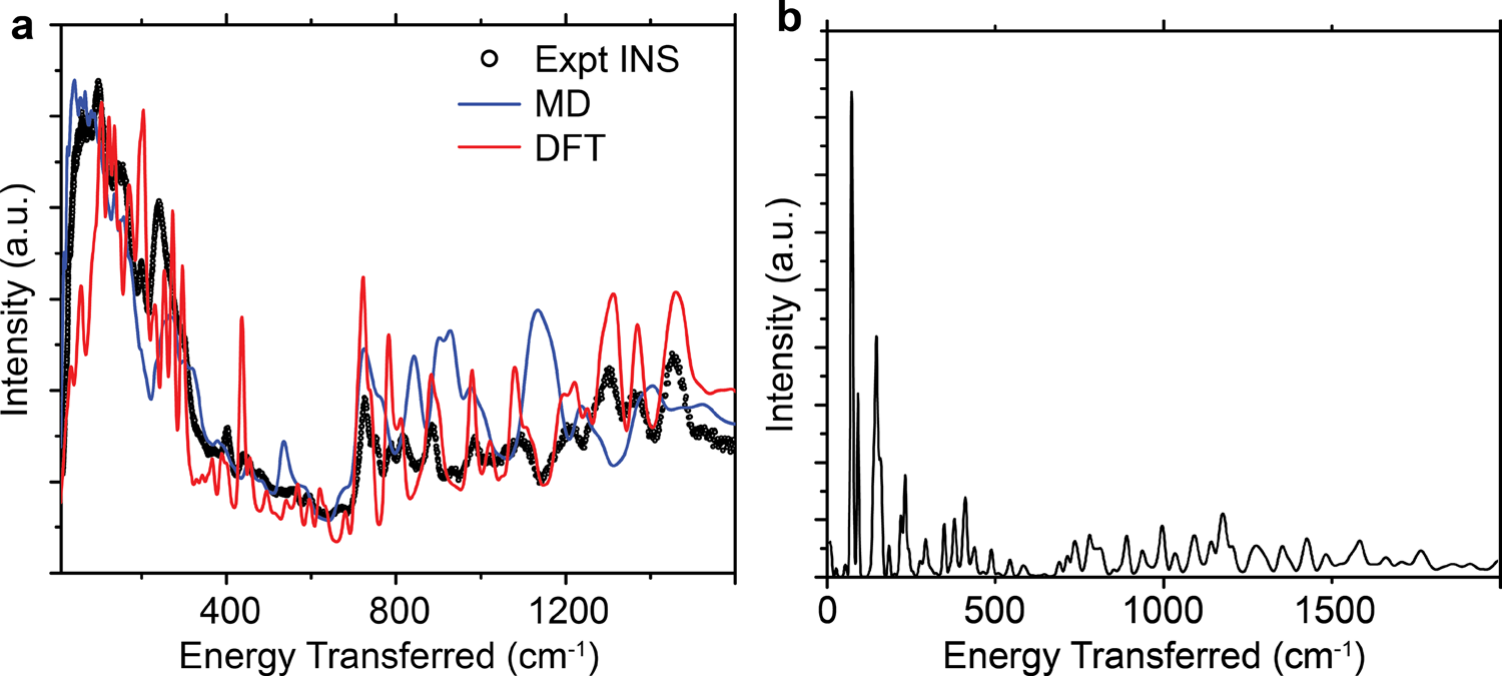
(**a**) Comparison between experimental INS spectrum, DFT simulated spectrum, and MD simulated spectrum. The MD spectrum was convolved by a Gaussian of width 0.01E, where E is the energy transfer. (**b**) The INS spectrum calculated from the normal modes of a supercell of the DFT structure using the MD forcefield is also plotted.

To demonstrate an improvement over the existing methodology for simulating INS spectra, we compare the MD simulated spectrum against INS data, and a full Brillouin zone DFT calculation of a single conformation of crystalline P3HT in Fig. 3a. The INS data in Fig. 3a is the result of subtracting 55% of a regiorandom P3HT INS spectrum (assumed to be completely amorphous) from the regioregular P3HT spectrum, meaning we are assuming that the P3HT sample is 45% crystalline, which agrees with prior literature^26^. At energies below 600 cm$^{-1}$, the MD simulation more accurately models the INS spectrum than the DFT simulation. Since prior literature has shown that DFT can accurately simulate INS spectra^13^, we can assume that the DFT spectrum is accurate for the chosen configuration, so the MD simulation is more accurate than the DFT simulation because it properly samples the relevant phase space of crystalline P3HT. This is clearly shown in Fig. 3b, where we plot the calculated INS spectrum using the normal modes obtained from the MD forcefield and a $1\times 2\times 2$ supercell of the DFT structure. We observe that the INS spectrum derived from the normal modes of the MD forcefield in Fig. 3b has much sharper features at low energy than the INS spectrum computed from our method in Fig. 3a, and these sharper features do not agree with the experimental data. In this way, we demonstrate that proper agreement between experiment and theory at low energy transfers requires the simulation of a structure that is more complex than small supercells of a crystalline motif of the polymer. In the intermediate energy range of 600–1600 cm$^{-1}$, the MD simulation shows poorer agreement with the experimental INS spectrum than the DFT simulation, indicating that DFT is better at accurately modeling the angle bends/wags, and bond stretch modes present in this energy range. In addition, we note that the experimental spectrum levels off beyond 1600 cm$^{-1}$, and there is decent agreement between the simulation and experiment in which the C–H stretch peak at $\sim$ 3000 cm$^{-1}$ is well parametrized in the forcefield, and the computed background scattering levels are accurate. The comparison between experiment and both simulations is available in Supplemental Information (Figure S5).

**Figure 4:**
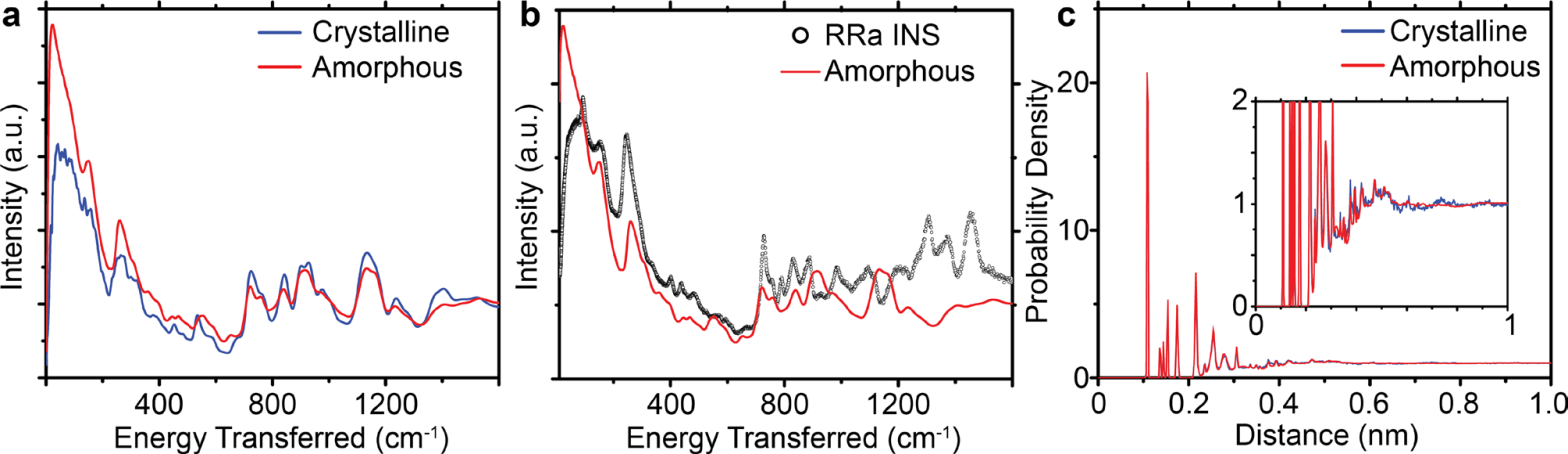
Comparison between MD simulated spectra for crystalline and amorphous P3HT materials. (**a**) Comparison between the MD simulated spectra of the crystalline and amorphous phases. (**b**) Comparison between experimental INS spectrum for regiorandom (RRa) P3HT, and the amorphous MD simulated spectrum. (**c**) Radial distribution functions comparing crystalline and amorphous regions of P3HT; the inset is the same plot expanded at low probability densities to demonstrate the differences between the two distributions. The MD spectrum was convolved by a Gaussian of width 0.01E, where E is the energy transfer.

For the amorphous volume of P3HT, we started from a box of 800 amorphous 20-mer chains annealed at 300 K, which was taken from Scherer et al.^27^. The average density of the structure was 1.04 g/cm$^3$, which reasonably agrees with prior literature values for the room temperature density of amorphous P3HT^29^ (1.09 g/cm$^3$). The details for the melting and annealing of the simulation box are in the Methods section. The simulated amorphous spectrum is compared against the crystalline simulated spectrum in Fig. 4a. There are significant differences between the two spectra at low energies, and the change between the two spectra is not consistent with the experimental INS spectrum of regiorandom (RRa) P3HT in Fig. 4b. The difference between the two simulated spectra can be explained by the difference in their densities: 1.04 g/cm$^3$ for the amorphous phase, and 1.08 g/cm$^3$ for the crystalline phase. This is exacerbated during the quench to 10 K, where the crystalline density increases to 1.16 g/cm$^3$, and the amorphous density stayed constant at 1.04 g/cm$^3$. The change in density causes an increase in the average intermolecular spacing that reduces the average van der Waals contribution to the force constants between molecules, and decreases the frequency of the dynamics that red-shifts the spectrum. In Figure S4 of the Supplemental Information, we compare the experimental INS spectra for RR and RRa P3HT, which shows that there is very little change at low energies. This means that the average intermonomer spacings are consistent between RR and RRa P3HT, indicating that the amorphous simulation is not sampling the correct morphologies. In Fig. 4c, we compare the radial distribution functions for the amorphous and crystalline simulations, and find that the morphologies appear similar using these functions, which indicates the sensitivity of INS spectra to changes in local morphology.

While it is tempting to attribute the disagreement between experiment and simulations to the sampled morphologies, it is entirely possible that some portion of the error comes from the quality of the empirical forcefield and its tranferability to low temperature dynamics. However, it is impossible to disentangle the impacts of morphology versus forcefield because we do not know how morphology changes upon lowering the temperature to 10 K in the various phases of P3HT. For instance, we do not have any information on the density of P3HT at cryogenic temperatures, so we do not have the information to determine the quality of our low-temperature morphologies. However, the large differences in simulated densities at low temperature, along with the quality of the agreement between the crystalline simulation and RR P3HT, indicates that the amorphous morphology is likely to be the dominant source of error.

To determine the quality of the agreement between the simulations and the experimental data, we use a least-squares error statistic weighted by the average experimental error. The equation for determining the error is given below,

24 $$\begin{aligned} E = \frac{1}{\sqrt{\langle \sigma ^2\rangle } } \sqrt{\frac{\sum _i^N \left( x_i - x_i^o\right) ^2}{N}} \end{aligned}$$

where $x_i$ and $x_i^o$ are the simulated intensities and experimental intensities at frequency *i*, respectively, and $\langle \sigma \rangle$ is the average experimental error of the spectrum.

**Table 1:** Weighted least squares error for the comparisons of the three simulations to the experimental data. E$_{low}$ and E$_{high}$ are the weighted least squares errors for the energy ranges 10 cm$^{-1}$–600 cm$^{-1}$ and 600 cm$^{-1}$–3500 cm$^{-1}$, respectively.

Simulation	E$_{low}$	E$_{high}$
Crystalline MD	0.210	0.371
Amorphous MD	0.337	0.284
Crystalline DFT	0.289	0.272

Table 1 compares the weighted least square error for each of the simulations. In the low energy region (10 cm$^{-1}$–600 cm$^{-1}$), the crystalline MD simulation more accurately represents the data than the other simulations. This quantitatively demonstrates that the presence of multiple morphologies in the larger MD simulation leads to a more accurate agreement with experimental data for low energy dynamics. In the high energy region (600 cm$^{-1}$–3500 cm$^{-1}$), the crystalline DFT simulation is the most accurate, which makes sense because the classical forcefield has not been modified specifically for the bond vibrations present in P3HT. When comparing the two MD simulations, we see that the amorphous simulation is significantly less accurate than the crystalline one, except in the high energy region. Upon inspection of the peaks $>600$ cm$^{-1}$ in Figs. 3 and 4, we observe that the reduction in error for the amorphous simulation comes from a broadening of the peaks, and not from any peak shifts.

## Discussion

We have demonstrated a novel method for computing INS spectra from classical molecular dynamics trajectories. Our new method is theoretically equivalent to more common quantum based normal mode approaches, while being more computationally scalable. We expect that the method becomes computationally limited for systems with extremely large numbers of atoms because the size of the velocity data files become challenging to process quickly. However, the atomic components of the dynamic structure factor do not depend on each other, which means we can use a smaller set of representative atoms to construct the dynamic structure factor to mitigate the computational load due to the dataset size. Other options for reducing the dataset size include coarse-graining the system prior to simulation, and/or using a low-pass filter on the velocity data. Despite these challenges, our approach allows for simulations of much larger systems than those studied in prior INS publications. As a result, this method opens the door to many research areas that can use INS as an analytical tool, but have not yet done so because the target molecules/materials are too complex to simulate using ab initio methods. Our complex example material is semicrystalline P3HT, and we were able to use our method, along with experimental INS data, to make a valuable, direct comparison between experimental and simulated low energy dynamics. Such a direct comparison with molecular dynamics simulations is not possible with other experimental techniques.

In our example, we demonstrated that the simulation of larger scale morphologies of P3HT using molecular dynamics yielded better agreement with experimental INS spectra than crystalline density functional theory simulations in the 10–600 cm$^{-1}$ energy range. In the range beyond 600 cm$^{-1}$, the DFT simulation is most accurate. The disagreement between the INS data and the MD simulated spectra at energies above 600 cm$^{-1}$ come from small errors in the bonded potential energy that are common in generic forcefields like OPLS-AA. Our analysis also revealed that the amorphous simulation was sampling the incorrect morphologies, which is the dominant contribution to the disagreement with the experimental RRa P3HT INS spectrum. To improve the quality of the agreement further, particularly for the crystalline simulation, the non-bonded parameters of the empirical forcefield of P3HT need to be optimized. We showed that changes in the amorphous density relative to the crystalline density caused large changes in the low frequency dynamics, demonstrating that the low energy region of simulated INS spectra is sensitive to morphological changes. Therefore, our method provides important information that describes the impact of the forcefield and the simulated non-equilibrium morphologies on low frequency dynamics, which is not accessible by any other combination of simulation and experiment.

Currently, the limits of this method are unknown. Perhaps altering the MD forcefield through force matching could extend the energy range for which this technique is useful. A study which uses force matching in conjunction with our new technique should be performed on a molecule which DFT is known to work particularly well and compared not only to DFT but to all common methods for computing an INS spectrum.

## Methods

Molecular dynamics simulations were performed using the Gromacs simulation package^30–32^ installed on a high-performance computing cluster at UC Davis. Crystalline P3HT simulations began as a slice of 16 chains with 20 monomers from a larger crystalline slab generated by Scherer et al.^27^, which were equilibrated at 300 K for 15 ns. We then lowered the control temperature of the thermostat to 10 K, and simulated in an NpT ensemble for 10 ns, allowing the kinetic temperature to reach equilibrium. We used a velocity-rescale thermostat^33^ with a time constant of 1 ps, and an anisotropic Parrinello–Rahman barostat^34^ with damping constants of 1 ps and $4.5\cdot 10^{-5}$ bar$^{-1}$ in all directions. For the production run in which we simulated the INS spectrum, the thermostat time constant was increased to 10 ps, we switched to an Andersen massive thermostat, we turned off the barostat, and fixed the lattice dimensions to their average values over the final 1 ns of the equilibration run. The 100 ps production runs recorded atomic velocities every 2 fs to properly record every vibrational mode in the material. We switched to the Andersen massive thermostat^35^ to properly decorrelate the atomic motions after 10 picoseconds, which ensured the success of our method. However, we ultimately found no dependence on the observed spectrum and the type of thermostat used; see SI section S4. If the time constant is decreased to 1 picosecond, then the thermostat interferes with the simulated INS spectrum, also shown in section S4. The NpT ensemble was not used for production runs because the barostat dampens the dynamics of the molecules (see SI section S4).

The amorphous structure for P3HT was also started from configurations generated by Scherer et al.^27^. We repeat some details here for clarity. For the amorphous region of P3HT, a box of 1600 20-mer chains in the crystalline configuration above was simulated at 750 K for 3.7 ns to randomize all configurations. The box was brought to 300 K by stepping down the temperature in 50 K increments and simulating for 200 ps. Once 300 K was reached, the box was annealed for 61 ns. The average density of the final structure at 300 K was 1.04 g/cm$^3$, which reasonably agrees with literature values^29^. From 300 K, the amorphous system was ramped down to 10 K over 75 ns at a constant rate in the NpT ensemble, and held there for 10 ns. We used an Andersen-massive thermostat with a time constant of 1 ps, and an anisotropic Parrinello–Rahman barostat with the same damping constants as the crystalline quench to 10 K. Then, we averaged the lattice parameters over the latter half of the 10 K NpT simulation to fix the lattice parameters for the 100 ps NVE production run, which we used to simulate the INS spectrum.

INS spectra were computed using a Python code developed by our group (available online^36^) utilizing mpi4py^37–39^ to parallelize the calculation. All convolution and correlation operations were performed via Fourier transforms within the Scipy package. The resolution function of the VISION instrument was assumed to be Gaussian with a width that scales with energy transfer as $\sigma = 1.21 + 0.01\Delta E$, where the units are in cm$^{-1}$, and $\Delta E$ is the energy transfer. We made this choice for the resolution function because this function led to a reasonable agreement between the simulated and measured widths of the C–H stretch mode at $\sim 3200$ cm$^{-1}$, and it has been reported that the resolution of the VISION spectrometer scales with the energy transfer in a similar way^17^. We plan to add instrument-specific resolution functions to the code as needed. We assumed that the energy of the scattered neutron that hits the detector is fixed at 32 cm$^{-1}$, which is typical of inverted neutron spectrometers like VISION^11^.

The INS spectra determined from DFT calculations were computed using a combination of VASP using the standard PAW pseudopotentials^40–43^, Phonopy^44^, and oCLIMAX tool provided by Oak Ridge National Lab^24^. Specifically, Phonopy was used to create structures with small atomic displacements (0.01 $\AA$) of a $2\times 2\times 2$ supercell of Config2 from a prior study^4^, and VASP computed the atomic forces for each perturbed structure. Then, the set of forces was passed back to Phonopy, and the phonons were computed for a $7\times 7\times 7$ k-point grid. Then, these phonon files were passed to the oCLIMAX tool, which converted the phonons into an incoherent INS spectrum.

The INS spectrum determined from the normal modes of MD calculations was computed very similarly to the INS spectra computed from DFT calculations. The finite displacement calculations were performed using Gromacs and the same forcefield used for the MD simulations. The structure was the same used in the DFT calculations. A $1\times 2\times 2$ supercell was prepared and optimized. Then Phonopy was used to calculate the vibrational modes and energies over a $5\times 5\times 5$ k-point sampling grid using the finite displacement method in which the atoms were perturbed by 0.01 Å. The vibrational modes and energies were converted to a simulated INS spectrum using the oCLIMAX. The phonon Brillouin zone was sampled densely enough to achieve convergence in the simulated INS spectrum.

## Supplementary Information

Supplementary Information.
