# Supplementary material for: Computing inelastic neutron scattering spectra from molecular dynamics trajectories
Source: Sci Rep. 2021 Apr 12;11:7938. doi: 10.1038/s41598-021-86771-5 (PMC8041884; doi:10.1038/s41598-021-86771-5)
Supplement: Supplementary file 1 — Supplementary Information. [file 41598_2021_86771_MOESM1_ESM.pdf]

Supplementary Information - Computing Inelastic Neutron Scattering Spectra from Molecular Dynamics Trajectories

Thomas F. Harrelson[1, 2], Makena Dettmann[3], Christoph Scherer[4], Denis Andrienko[4], Adam J. Moulé[1], and Roland Faller[1,*]

[1]Department of Chemical Engineering, University of California - Davis, 1 Shields Ave, Davis, CA 95616, USA
[2]Molecular Foundry, Lawrence Berkeley National Laboratory, 1 Cyclotron Road, Berkeley, CA 94720, USA
[3]Department of Materials Science and Engineering, University of California - Davis, 1 Shields Ave, Davis, CA 95616, USA
[4]Max Planck Institute for Polymer Research, Ackermannweg 10, 55128 Mainz, Germany
[*]rfaller@ucdavis.edu

1 Normal mode decomposition of Molecular Dynamics Trajectory

The aim of this section is to explore the physical meaning behind eqs. 3-6 in the main text. We start by postulating the existence of a set of j normal modes in the system, which generates an exact dynamical description of the atomic displacements according to,

$$u_i(t) = c_{ij}u_j(t) \tag{S1}$$

where $u_i(t)$ is the displacement of atom i at time t, $u_j(t)$ is the displacement along the normal mode coordinate j, and c_{ij} is the unitary transformation matrix that connects the atomic and normal mode descriptions. In the harmonic approximation, the transformation matrix is time-independent, so taking the time derivative of both sides reproduces eq. 3 in the main text. With this in mind, we take the discrete Fourier transform of $v_i(t)$,

$$v_i(t_n) = \frac{1}{N}\sum_{j=0}^{N-1} V_i(\omega_j)e^{i\omega_j t_n} \tag{S2}$$

We identify ω_j as $2\pi j/t_{total}$ and t_n as $n\Delta t$, where t_{total} is the length of the MD simulation, Δt is the timestep of the simulation, j is an integer ranging from 0 to $N-1$, and n is a similar integer. This identification puts the discrete Fourier transform in a more usual form:

$$v_i(t_n) = \frac{1}{N}\sum_{j=0}^{N-1} V_{ij}e^{i2\pi jn/N} \tag{S3}$$

where N is the number of time-steps in the simulation. Since $v_i(t)$ must be a real number, the Fourier transform must be Hermitian, which means $V_{ij} = V^*_{i,N-j}$. Thus, we can write the transform as,

$$v_i(t_n) = \frac{1}{N}\left(V_{i0} + \sum_{j=1}^{N/2} V_{ij}e^{i2\pi jn/N} + V^*_{ij}e^{-i2\pi jn/N}e^{i2\pi n}\right) \tag{S4}$$

where the right-most exponential is always 1. If we write V_{ij} as $|V_{ij}|e^{i\delta_j}$, then we can simplify the transform to,

$$v_i(t_n) = \frac{1}{N}\left(V_{i0} + \sum_j 2|V_{ij}|\cos(2\pi jn/N + \delta_j)\right) \tag{S5}$$

where we identify V_{i0} as the drift in the simulation box, which we set to zero. If we insert definitions of ω_j and t_n into the cosine argument, we get,

$$v_i(t_n) = \frac{1}{N}\sum_j 2|V_{ij}|\cos(\omega_j t_n + \delta_j) \tag{S6}$$

Comparing to eq. 3 and eq. 4 in the main text, we see that,

$$v_i(t_n) = \sum_j c_{ij} V_j \cos(\omega_j t_n + \delta_j) = \frac{1}{N} \sum_j 2|V_{ij}| \cos(\omega_j t_n + \delta_j) \tag{S7}$$

which means that our mode expansion in eq. 3 of the main text is analogous to the Fourier transform of the time series of the velocities of atom i. To be clear, this is an approximation in the general case, which becomes exactly true when all vibrational eigen-modes have non-degenerate frequencies. In this case, the Fourier transform will be able to pick up the dynamics of each mode independently. If there are modes that have degenerate eigen-frequencies, then constructive and destructive interference is possible between the degenerate Fourier components of the dynamics. As mentioned in the main text, we assume that each of the degenerate modes are decorrelated, which means the expectation value of the phase ($\langle \delta_j \rangle$) is zero. We can see the impact of non-zero phases by writing out a set of degenerate modes at frequency ω_j,

$$2|V_{ij}| \cos(\omega_j t_n + \delta_j) = \sum_d^{N_{degen}} c_{id} V_d \cos(\omega_j t_n + \delta_d) \tag{S8}$$

where we observe that the degenerate modes can interfere with each other, and the interference is determined by the relative values of δ_d. In a perfectly harmonic system, this is a problem, because the Fourier coefficients lose the information of the relative phases of the degenerate modes. However, we avoid this problem by noting that the system is weakly anharmonic, which decorrelates the modes, and we also use a weak thermostat to decorrelate the modes manually. In this case, the phase of each degenerate mode has a finite correlation time (e.g. $\langle \delta_d(0)\delta_d(t) \rangle \sim e^{-t/\tau}$). If the total MD simulation time is much longer than the largest correlation time, then the Fourier transform becomes similar to an average over several Fourier transforms of randomized initial conditions (within the correct thermodynamic ensemble). The impact of this average is most easily observed when considering the Fourier transform of the velocity autocorrelation function. If we label the left-hand side of eq. S8 as $v_i(t)$, we find that its power spectrum is,

$$F\{\langle v_i(0) v_i(t) \rangle\} = \left| \sum_d^{N_{degen}} \frac{c_{id} V_d}{2} e^{i\delta_d} (\delta(f - f_j) + \delta(f + f_j)) \right|^2 \tag{S9}$$

where $f_j = \omega_j/(2\pi)$. Expanding the square leads to,

$$F\{\langle v_i(0) v_i(t) \rangle\} = \sum_{d,d'} \frac{c_{id} c_{id'} V_d V_{d'}}{4} e^{i(\delta_d - \delta_{d'})} (\delta(f - f_j) + \delta(f + f_j)) \tag{S10}$$

We see that the $d = d'$ terms in the sum are always non-zero, and the $d \neq d'$ terms carry a phase $e^{i(\delta_d - \delta_{d'})}$ that averages to zero if we average over a series of random phases as discussed above. Thus, we obtain an equation for degenerate modes in terms of the Fourier coefficients of $v_i(t)$ as,

$$\frac{|V_{ij}|^2}{N^2} (\delta(f - f_j) + \delta(f + f_j)) = \sum_d \frac{c_{id}^2 V_{id}^2}{4} (\delta(f - f_j) + \delta(f + f_j)) \tag{S11}$$

We remind the reader that V_{ij} is the $j-$th Fourier component of the velocity function of atom i. As a result, we see that the relation that the square Fourier component $|V_{ij}|^2$ is the proportional to the sum of the contributions of all modes that are degenerate at frequency ω_j.

To see how eq. 5 in the main text relates to the numerical objects used in the calculation, we insert the relationships between the mode expansion parameters and the Fourier transform into eq. 5,

$$\sum_j \frac{|c_{ij}|^2 V_j^2}{4} \delta(f - f_j) = \sum_j \frac{|V_{ij}|^2}{N^2} \delta(f - f_j) \tag{S12}$$

However, in a discrete basis, the delta function changes by $\delta(f - f_j) \rightarrow N\Delta t \delta_{jj'}$ where $\delta_{jj'}$ is the Kronecker delta, and j' becomes the free index of f in a discrete representation. Therefore, we can write the above equation as,

$$\sum_{j'} \frac{|c_{ij'}|^2 V_{j'}^2}{4} \delta(f_j - f_{j'}) = \frac{|V_{ij}|^2 \Delta t}{N} \tag{S13}$$

where we have changed the summed index on the left-hand side to j' to explicitly write the free index as j. The above equation demonstrates how eq. 5 in the main text is related to Fourier transforms of the atomic velocities, which shows how the approach in the main text is implemented in the code. Note that the left-hand side of above equation differs from eq. 5 in the main text because of the relation: $2\pi\delta(\omega-\omega_j)=\delta(f-f_j)$.

We derive eq. 8 in the main text from eqs. 5-7 by first rearranging eq. 7 into,

$$\mu_j = \frac{2k_B T}{V_j^2} \tag{S14}$$

which we insert into eq. 6 to get,

$$u_{ij} = \frac{\hbar |c_{ij}|^2 V_j^2}{4\omega_j k_B T}. \tag{S15}$$

Multiplying both sides by $\delta(f-f_j)$, and summing over the index j, we get,

$$\sum_j u_{ij}\delta(f-f_j) = \sum_j \frac{\hbar |c_{ij}|^2 V_j^2}{4\omega_j k_B T}\delta(f-f_j) \tag{S16}$$

where we identify the components of the velocity power spectrum from eq. 5 in the main text on the right-hand side of the above equation. Substituting eq. 5 in the above equation produces eq. 8 from the main text. We also see that the above equation can be written in terms of Fourier coefficents through eq. S13, which is how these equations are implemented in the code.

2 Deriving Intermediate Scattering Function from Velocity Correlations

We start by defining the convolution operation between $\overline{\overline{B}}$ and any arbitrary function as an operator, which allows the general description of any overtone:

$$\overline{\overline{B}}_{n\leftarrow 0}(\omega) = \left(\hat{C}\right)^n \overline{\overline{B}}(\omega) \tag{S17}$$

$$\hat{C}\overline{\overline{B}}(\omega) = \int \overline{\overline{B}}(\omega')\overline{\overline{B}}(\omega-\omega')d\omega' \tag{S18}$$

The convolution operator is written into the scattering law as a power series of convolution operations. That power series simplifies to the exponential of the convolution operator:

$$S_i(\vec{q},\omega) = \sigma_i e^{-\int F_i(\omega)d\omega} e^{\hat{C}}\delta(\omega) \tag{S19}$$

$$F_i(\omega) = \frac{\hbar}{2\omega k_B T}\left|\vec{q}\cdot\vec{v}_i(\omega)\right|^2 = \left|\vec{q}\cdot\vec{r}_i(\omega)\right|^2 \tag{S20}$$

where $\vec{v}_i(\omega)$ is the Fourier transform of the classical velocity trajectory of atom i. By taking the Fourier transform of eq S19 we obtain:

$$I_i(\vec{q},t) = \sigma_i e^{-\int F_i(\omega)d\omega} e^{f_i(t)} \tag{S21}$$

$$f_i(t) = \langle[\vec{q}\cdot\vec{r}_i(0)][\vec{q}\cdot\vec{r}_i(t)]\rangle \tag{S22}$$

Using the identity of thermal averages,

$$e^{\langle A^2\rangle}e^{\langle AB\rangle} = \langle e^A e^B\rangle \tag{S23}$$

we show that eq S21 is equivalent to,

$$I_i(\vec{q},t) = \sigma_i\langle e^{-i\vec{q}\cdot\vec{r}_i(0)} e^{i\vec{q}\cdot\vec{r}_i(t)}\rangle \tag{S24}$$

which is the usual form of the intermediate scattering function for atom i, proving that our approach is consistent with any other forms of INS theory that begin from the known form of the intermediate scattering function in eq S24.

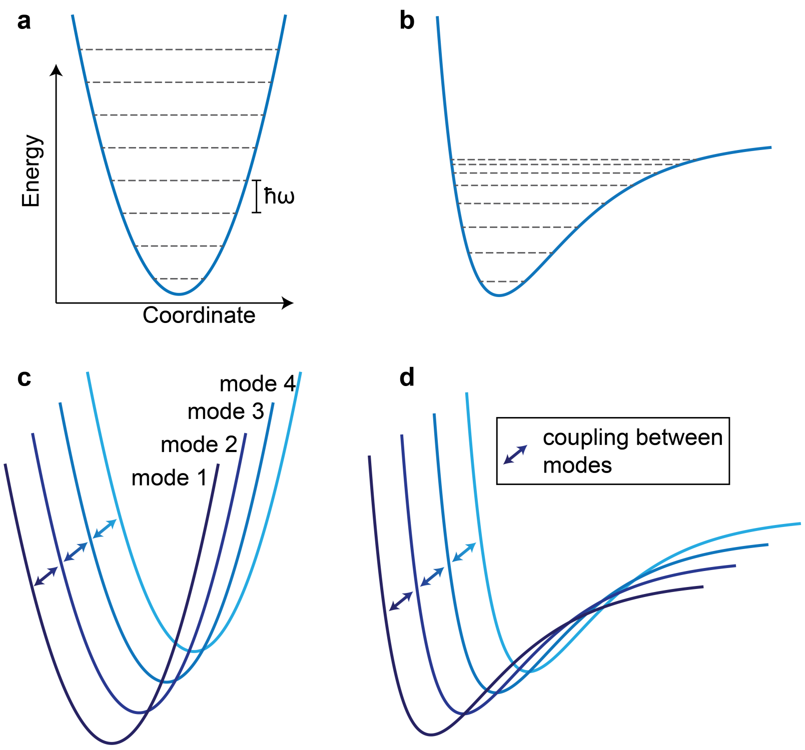

Figure S1. Schematic of harmonic and anharmonic potential energy surfaces. (a) Typical harmonic potential with quantum energy levels. (b) Anharmonic potential energy surface of a Morse potential with quantum energy levels. (c) A system of harmonic potential energy surfaces coupled together by anharmonicities. (d) A system of anharmonic potential energy surfaces that are coupled together.

3 Correcting Anharmonic Effects in INS Spectra Using Classical Dynamics

One characteristic feature of anharmonic quantum oscillators is that they have decreasing energy-level spacings with increasing quantum number as shown in Figure S1b. In classical trajectories of isolated anharmonic oscillators, the power spectrum contains overtones in addition to the peak at the fundamental frequency. This differs from harmonic quantum oscillators, which always have the same energy difference between adjacent energy levels (Figure S1a), and the classical power spectrum only has one peak at the fundamental frequency. We took advantage of this property in our formulation of neutron scattering spectra with the usage of Fourier convolutions to create overtone spectra. These convolutions implicitly assume a harmonic formulation because they create a set of peaks that are equally spaced in energy. While isolated oscillators can have anharmonic potential energy surfaces, it is also possible that anharmonicities couple two different harmonic modes together as in Figure S1c. The coupling between these states cause the transfer of energy between them, which means, when using phonon terminology, that the phonon populations diffuse between the different states. Once a phonon is created in an initial state, then it has a specific lifetime before it diffuses to a different state. This behavior manifests as a broadening of peak widths in the classical power spectrum. The coupling between different modes also decreases the energy level spacings. The most general description of anharmonicities in the solid state include coupled anharmonic potential energy surfaces as seen in Figure S1d. Since we seek to account for anharmonic effects in the INS spectra, we seek to augment the convolutions to account for the changes in energy level spacing.

We find a starting description of anharmonic oscillators in Atkins[1], in which the energy levels are given by a power series of the quantum number of the harmonic oscillator levels. If we truncate it to the lowest anharmonic order,

$$E_n = \hbar\omega\left[\left(n+\frac{1}{2}\right)+x_e\left(n+\frac{1}{2}\right)^2\right] \tag{S25}$$

then we calculate the difference between energy levels to be,

$$E_n - E_{n-1} = \hbar\omega\left[1-2nx_e\right] = \hbar\omega + \Delta E_i^{(2)} \tag{S26}$$

We can see how the energy level spacing decreases with increasing n. We ignore the condition that the energy level difference becomes negative at large values of n because the important spectral features occur at low n, and the anharmonicity constant is expected to be small, particularly at low temperatures.

We can correct our spectra by performing additional Fourier convolutions that shift the energy level differences. We define a function $k_i(\omega)$ that represents the deviation from the harmonic energy level differences of the ith level. Using this set of functions, we can compute the nth overtone spectrum as,

$$f_n(\omega) = \delta(\omega - k_1(\omega)) * f_1(\omega) * \delta(\omega - k_{n-1}(\omega)) * f_{n-1}(\omega) \tag{S27}$$

which is defined to be consistent with eq S26 above. This is not formally exact, and the approximation breaks down when there are multiple modes at the same frequency ω that have different energy level shifts. Thus, $k_i(\omega)$ represents the average energy shift for modes at frequency ω. We need to define $k_i(\omega)$ in terms of quantities calculated from classical trajectories.

We start by assuming that the quantum mechanical energy differences can be described by second order perturbation theory,

$$E_i = E_i^{(0)} + E_i^{(1)} + E_i^{(2)} \tag{S28}$$

$$E_i^{(2)} = \sum_{j \neq i} \frac{\langle i|V|j\rangle\langle j|V|i\rangle}{E_i^{(0)} - E_f^{(0)}} \tag{S29}$$

where $E_i^{(1)}$ is equal to zero for the lowest order anharmonic operator ($\Gamma_{i,j,k}(\hat{a}_i + \hat{a}_i^\dagger)(\hat{a}_j + \hat{a}_j^\dagger)(\hat{a}_k + \hat{a}_k^\dagger)$, where $\hat{a}_i$ and $\hat{a}_i^\dagger$ are the annihilation and creation operators for mode i). Computing $k_i(\omega)$ is equivalent to finding the set of $E_i^{(2)}$ for all modes in the system.

The anharmonicities manifest as decay rates of periodic motions, which broadens peaks in the power spectrum (typically with a Lorentzian lineshape). If we model the classical dynamics as a set of decaying harmonic oscillators (as in the main text), then we can compute a set of decay constants (k_j) that correspond to frequencies (ω_j).

$$\langle v_i^2 \rangle = \sum_j V_{ij}^2 e^{i\omega_j t - k_j t} \tag{S30}$$

We aim to relate the relaxation rates (k_j) of the classical simulation to the second order energy correction ($E_j^{(2)}$) found by perturbation theory to correct the overtones. We start with a first order correction to the time dependent wavefunction projected onto an arbitrary final state (assuming the initial state is the vibrational ground state for all oscillators).

$$c_f(t) = \delta_{fi} - \frac{i}{\hbar}\int_0^t \langle f|V|i\rangle e^{-i\omega_{fi}t'}dt' \tag{S31}$$

where c_f is the wavefunction coefficient for state f. In this equation, V is the anharmonic operator truncated to lowest order. From this, we can find the time-dependent population density of state f.

$$|c_f(t)|^2 = P_f(t) = \frac{4V_{fi}^2}{\hbar^2\omega_{fi}^2}\sin^2\left(\frac{\hbar\omega_{fi}t}{2}\right) \tag{S32}$$

where V_{fi} is the off-diagonal matrix element connecting states i and f. From this equation, we find that the time scale of the conversion from state i to f is π/ω. Now, we calculate the population transfer rate over this time scale. Defining the rate as,

$$R_f(t) = \frac{dP_f(t)}{dt} \tag{S33}$$

We can compute the time average from 0 to π/ω for each rate into all the possible final states. Since the rates can be combined additively to find the total rate leaving state i, we can define the total rate of disappearance from state i as,

$$\langle R_i(t)\rangle_{t=0}^{t=\pi/\omega_{fi}} = -\sum_f \langle R_f(t)\rangle_{t=0}^{t=\pi/\omega_{fi}} \tag{S34}$$

Carrying out the algebra we find a natural relationship between the average rate out of state i and $E_i^{(2)}$:

$$\langle R_i \rangle = -\sum_f \frac{4\langle i|V|f\rangle\langle f|V|i\rangle}{\pi\hbar\left(E_f - E_i\right)} = \frac{4E_i^{(2)}}{\pi\hbar} \tag{S35}$$

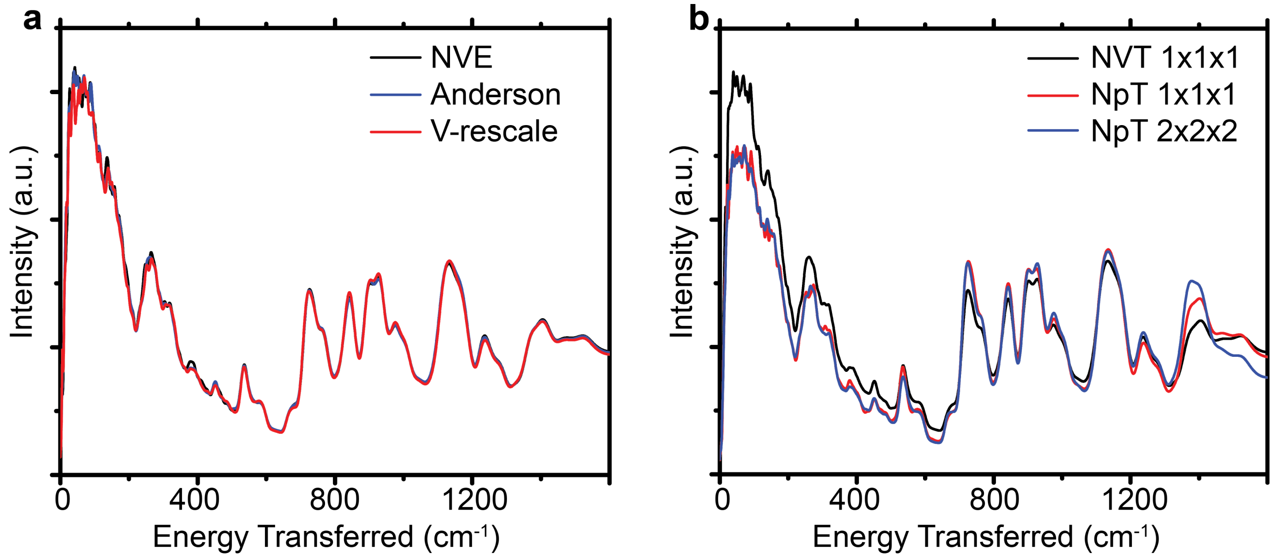

Figure S2. (a) Comparison between simulated INS spectra computed with no thermostat (NVE ensemble), an Andersen-massive thermostat, and a velocity-rescale thermostat (each with $\tau = 10$ ps). (b) Comparison between simulated INS spectra computed from a $1 \times 1 \times 1$ cell with no barostat (NVT ensemble), a $1 \times 1 \times 1$ cell with a Parrinello-Rahman barostat, and a $2 \times 2 \times 2$ cell with the same barostat.

This equation provides the quantum-classical correspondence that we need to correct for anharmonicities in our simulated INS spectra. However, since the dynamics were computed classically, the mode energies may be below the zero-point energy, which leads to inaccuracies in the observed anharmonic transfer rates. In addition, the rates computed from classical dynamics must be consistent with the thermodynamic ensemble of the simulation (we assume a NVT ensemble), so the RHS of eq S35 is the thermal average over the set of $E_i^{(2)}$. It may be sufficient to simply ignore the thermodynamic average, and assume that the anharmonic energy perturbations are equal the rates computed from low-temperature classical MD simulations. If one desires to correct for the thermodynamic average, then we suggest to perform multiple MD simulations at different temperatures, which will approximate the functional dependence of the set of rates on temperature, $\{R_i(T)\}$. Finally, interpolate each of these functions to find the rate when the temperature is equal to $\frac{\hbar\omega}{k_B}$, where k_B is the Boltzmann constant.

4 Impact of Thermostats and Barostats

For simulations of inelastic neutron scattering experiments, the choice of whether to use thermostats and barostats is difficult. Thermostats unphysically alter velocities on characteristic timescales, which can impact the observed velocity correlation functions use in our calculation of INS spectra. Barostats can also impact the observed velocity correlation functions, because the volume fluctuations also cause changes in interatomic distances, which impact potential energies and consequently the atomic dynamics. For both barostats and thermostats, we chose large time constants ($\tau = 10$ ps), to minimize the impact on computed spectra; a 10 ps timescale corresponds to a frequency of ~ 3 cm^{-1}, which is small enough such that it minimally impacts our computed spectra.

We start by testing the impact of different thermostats (each with $\tau = 10$ ps) in Figure S2a. In the main text, we chose to incorporate an Andersen-massive thermostat because our formalism required that all degenerate oscillators (degenerate meaning oscillating at the same frequency) be completely decorrelated. We chose to test a velocity-rescale, Andersen-massive, and no thermostat (NVE ensemble) in Figure S2a, and we see very little impact from both the presence of the thermostat, and type of thermostat used. The reason for choosing the two different thermostats is to show that complete decorrelation of all oscillators is not necessary at this scale; an Andersen-massive thermostat ensures complete oscillator decorrelation on the time scale τ, whereas the velocity-rescale thermostat will cause a decay of oscillator correlations on the time scale τ. If the phase of the independent oscillators in the simulation were heavily correlated, then there would be differences between peak heights computed from different thermostats (or no thermostat). The simulation results suggest that the oscillator phases are randomized by the large number of degenerate oscillators, or by anharmonicities present in the form of the classical forcefield. Each oscillator randomly starts at a phase between $-\pi$ to π, which averages to 0 if there are many oscillators at the same frequency (law of large numbers). Since the simulation contains sixteen 20mer polymer chains, there are 320 identical chemical

groups, which is likely large enough to randomize the oscillator phases.

The impact of barostats may be less controllable than thermostats, but they may play a subtly important role in dynamical systems. In Figure S2b, we show the impact of using a Parrinello-Rahman barostat on the computed INS spectra. We see that the barostat dampens the low frequency modes in the system. To test if this effect depends on the simulation box size, we computed the INS spectrum for an NPT simulation of a $2 \times 2 \times 2$ box of polymer chains (128 chains), and we see very little change between the computed spectra. This means that the lattice coordinates are coupled to the low energy modes of the molecules in the system, which is somewhat expected. However, the question of whether to use a barostat or not remains unanswered. It is important to note that a real crystalline material has phonon modes with wavelengths larger than the lattice dimensions used here, which are completely omitted from our simulations. This means that the barostat may mimic the action of these long range phonons, and thus may be physically appropriate to use for computing INS spectra. However, we did not choose to use a barostat in the main text because the mode dampening effect is strongly modulated by the compressibility value, which is unknown for P3HT. For our NpT simulations, we assumed that the compressibility of P3HT is $4.5 \cdot 10^{-5}$ bar^{-1}, which is the same as water at 300 K and 1 atm.

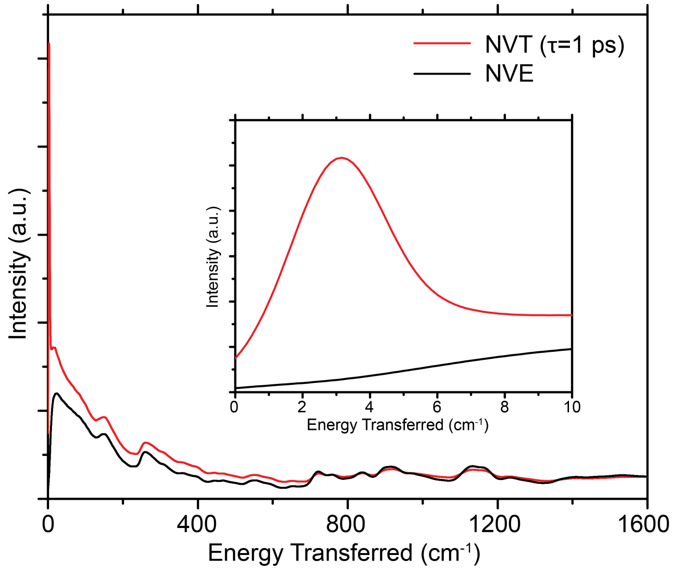

Figure S3. Comparison between two amorphous simulated INS spectra; one with a velocity-rescale thermostat (1 picosecond time constant), and one without a thermostat. The inset expands the spectra at extremely low energies, revealing the position of a tall peak at ~ 3 cm^{-1}.

We also compared the impact of the time constant of the thermostat on observed spectra. In Figure S2, the time constant was set to 10 picoseconds. In Figure S3, we demonstrate that a time constant of 1 picosecond using a velocity-rescale thermostat contributes a large, unphysical spike at ~ 3 cm-1, which is eliminated when the thermostat is turned off. Interestingly, the frequency of an oscillator with a period of 1 picosecond is approximately 3 cm^{-1}. This is evidence that the thermostat actually has a measurable impact on the simulated INS spectrum. Going further, this implies that the thermostat contributes to the correlated dynamics in the system, which is somewhat unexpected because the thermostat acts stochastically on the system.

5 Experimental Inelastic Neutron Scattering Comparisons

In Figure S4, we compare the INS spectra for pristine P3HT, and P3HT doped with F4TCNQ at a 4% mole ratio. We see that the energies of the peaks do not change, only the heights of the peaks. This is important to show because the pristine measurement was performed during a different measurement cycle than the doped and regiorandom (RRa) measurements. Between the two measurement cycles, the VISION spectrometer was slightly modified, such that the two measurements are not directly comparable. The changes in peak heights in Figure S4a is the manifestation of the instrument changes. Ideally, we would like to directly compare RRa P3HT with pristine, undoped P3HT, but this is not a fair comparison. Since the peaks for 4% doped P3HT are at the same energies as pristine P3HT, we use it as a surrogate, while implicitly assuming that the changes in peak heights are completely due to the changes in instrumentation. As a result, we compare RRa P3HT with 4% doped P3HT to demonstrate the differences between the amorphous and semicrystalline phases of P3HT.

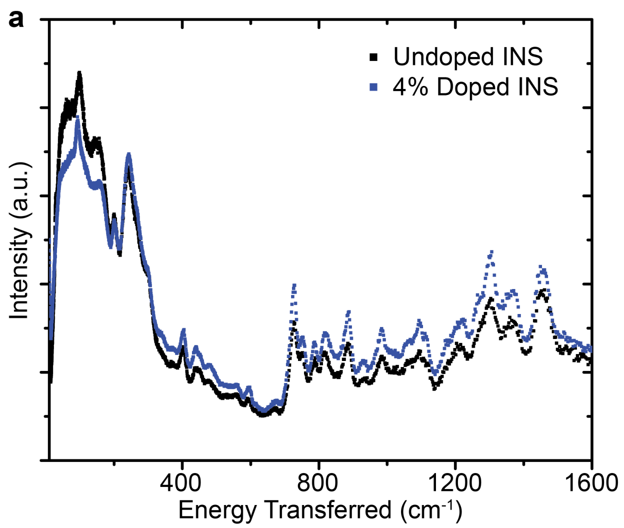

Figure S4. (a) Comparison between experimental INS spectra for undoped and 4% doped P3HT with F4TCNQ. (b) Comparison between regiorandom (RRa) P3HT and 4% doped P3HT.

6 MD versus DFT Comparison

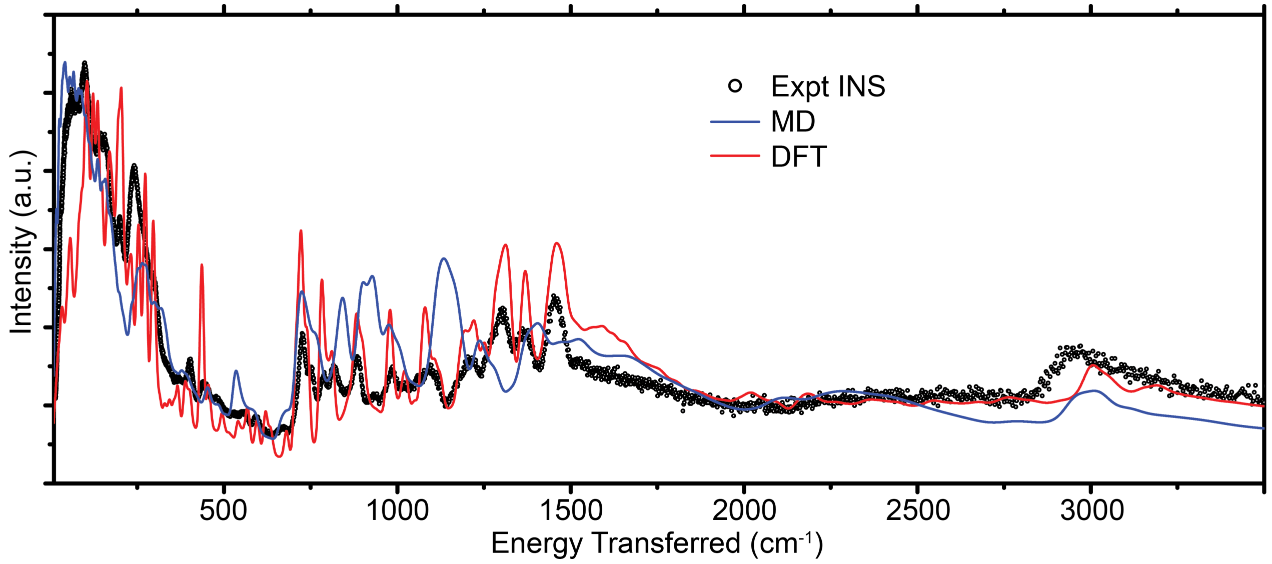

Figure S5. Comparison between INS data and both MD/DFT simulations over the full energy range (10 cm^{-1}-3500 cm^{-1})

In Figure S5 we show the comparison between the calculated INS spectrum derived from MD and DFT simulations over the full energy range. In the region between 1600 cm^{-1}–3000 cm^{-1}, there are no observed peaks, but the background signal comes from the overtones of the spectra. We show that the INS spectrum computed from MD displays the proper scatter signal strength in this region, which demonstrates that our methodology is accurately treating the overtone contributions to INS spectra.

References

1. Atkins, P. & de Paula, J. *Physical Chemistry for the Life Sciences* (W.H. Freeman and Company, 2006).
